# Efficacy of Vinflunine for Patients with Metastatic Urothelial Cancer after Immune Checkpoint Inhibitor Pretreatment—A Retrospective Multicenter Analysis

**DOI:** 10.3390/cancers14122850

**Published:** 2022-06-09

**Authors:** Felix Riedel, Mara Münker, Florian Roghmann, Johannes Breyer, Marco J. Schnabel, Maximilian Burger, Danijel Sikic, Thomas Büttner, Manuel Ritter, Kiriaki Hiller, Felix Wezel, Christian Bolenz, Friedemann Zengerling

**Affiliations:** 1 Department of Urology, University Hospital Ulm, 89081 Ulm, Germany; felix.wezel@uniklinik-ulm.de (F.W.); christian.bolenz@uniklinik-ulm.de (C.B.); friedemann.zengerling@uniklinik-ulm.de (F.Z.); 2 Department of Urology, Marien Hospital, Ruhr-University Bochum, 44625 Herne, Germany; maraanna.muenker@elisabethgruppe.de (M.M.); florian.roghmann@elisabethgruppe.de (F.R.); 3 Department of Urology, Caritas Hospital St. Josef, University of Regensburg, 93053 Regensburg, Germany; johannes.breyer@klinik.uni-regensburg.de (J.B.); mschnabel@csj.de (M.J.S.); mburger@caritasstjosef.de (M.B.); 4 Department of Urology and Pediatric Urology, University Hospital Erlangen, Friedrich-Alexander-University Erlangen-Nuremberg, 91054 Erlangen, Germany; danijel.sikic@uk-erlangen.de; 5 Department of Urology, University Hospital Bonn (UKB), 53127 Bonn, Germany; thomas.buettner@ukbonn.de (T.B.); mritter@ukbonn.de (M.R.); 6 National Center for Tumor Diseases (NCT) Heidelberg, 69120 Heidelberg, Germany; kiriaki.hiller@med.uni-heidelberg.de

**Keywords:** metastatic urothelial carcinoma, bladder cancer, immune checkpoint inhibition, immunotherapy, vinflunine, chemotherapy

## Abstract

**Simple Summary:**

With the introduction of immune checkpoint inhibitors (ICI) in recent years, the treatment landscape of metastatic urothelial cancer has undergone a substantial transformation. Nevertheless, disease progression after prior platinum-based chemotherapy and ICI pretreatment remains a challenging clinical situation with little evidence for following therapeutic options. The aim of this multicenter analysis was to examine the efficacy of the vinca alkaloid vinflunine after previous ICI therapy. In our cohort, post-ICI patients showed an overall response rate (ORR) of 22.4% compared to 15.6% within ICI-naïve patients (*p* = 0.451), and the clinical benefit rate (CBR) was 51.0% vs. 25.0% (*p* = 0.020), respectively. Post-ICI patients showed longer OS (8.78 vs. 5.72 months; *p* = 0.467) and longer PFS (3.09 vs. 2.14 months; *p* = 0.105). Our analysis demonstrates the clinical activity of vinflunine in a third- or later-line post-ICI setting, and the therapeutic benefit may be considerably higher than demonstrated in previous studies.

**Abstract:**

Background: Immune checkpoint inhibitors (ICI) are standard of care in patients with metastatic urothelial carcinoma (mUC) ineligible for cisplatin, and as second-line therapy after platinum-based chemotherapy. To date, few data exist about the efficacy of the former second-line chemotherapeutic agent vinflunine after the failure of sequential platinum-based chemotherapy and ICI treatment. The aim of this analysis was to examine the efficacy of vinflunine in a post-ICI third- or later-line setting. Methods: In this retrospective German multicenter study, data of mUC patients treated with vinflunine were reviewed in six centers between February 2010 and December 2021. All of the 105 included patients had radiologic progression after first-line platinum-based chemotherapy. The objective was to describe the efficacy of vinflunine in terms of overall response rate (ORR), clinical benefit rate (CBR), overall survival (OS), and progression-free survival (PFS) for post-ICI and ICI-naïve patients, respectively. Results: In our cohort, 61 patients (58.1%) had preceding immunotherapy before vinflunine administration, and 44 patients (41.9%) were ICI-naïve. Patients with ICI pretreatment showed an ORR of 22.4% compared to 15.6% within ICI-naïve patients (*p* = 0.451), and CBR was 51.0% vs. 25.0% (*p* = 0.020), respectively. Post-ICI patients showed longer OS (8.78 vs. 5.72 months; *p* = 0.467) and longer PFS (3.09 vs. 2.14 months; *p* = 0.105). Conclusion: This analysis supports the sequential use of vinflunine in post-ICI patients since the vinca-alkaloid retains a measurable clinical activity in these heavily pretreated patients. The therapeutic benefit may be higher than demonstrated in previous studies.

## 1. Introduction

With the introduction of immune checkpoint inhibitors (ICI) in recent years, the treatment landscape of metastatic urothelial carcinoma (mUC) had undergone a substantial transformation. In patients eligible for cisplatin-based chemotherapy, ICI is the current standard of care for maintenance therapy or second-line treatment, resulting in an improved overall survival (OS) exceeding two years after diagnosis of metastatic disease [1]. Patients not eligible for cisplatin can receive ICI as a first-line treatment depending on PD1/PD-L1 expression [2]. Although ICI therapy has improved the prognosis of mUC and has a more favorable side effect profile than chemotherapy, most patients will not experience long-term benefits [3,4], while a small percentage will experience rapid progression [5]. For the overwhelming majority mUC remains an incurable disease and accounts for approximately 200,000 deaths every year [6,7].

Disease progression after prior platinum-based chemotherapy and ICI therapy is a challenging clinical situation. Regarding the question of third-line therapy initiation, limited chances of clinical benefits of a later-line therapy must be balanced against burdensome adverse events in heavily pretreated patients [8].

In this setting the vinca-alkaloid vinflunine represents a therapeutic option approved by the European Medicines Agency (EMA) in 2009 after the pivotal randomized phase III trial by Bellmunt et al. showed a discrete OS advantage in the per protocol analysis compared to best supportive care [9]. However, lacking overall survival (OS) benefit in the intention to treat (ITT) analysis and a rather low response rate of 8.6% are the reasons why U.S. Food and Drug Administration (FDA) approval was not obtained [10] and vinflunine is not recommended by National Institute for Health and Clinical Excellence [11]. A real-world data analysis by Bamias et al. showed superior efficacy with an overall response rate (ORR) of 18% and a tolerable safety profile of vinflunine in an unselected mUC population of 797 patients [10]. To date, little evidence exists about the use of vinflunine as a third-line agent after pretreatment with platinum-based chemotherapy and checkpoint inhibition [12].

The aim of this national multicenter analysis was to examine the efficacy of the vinca-alkaloid vinflunine after previous ICI therapy (post-ICI cohort) and to compare results with patients without ICI pretreatment (ICI-naïve cohort).

## 2. Materials and Methods

We performed a retrospective analysis with patients from a total of 6 German tertiary referral centers. We included adult patients with histologically confirmed mUC originating from the bladder or the upper urinary tract who received treatment with vinflunine after having experienced radiological progression after previous platinum-based chemotherapy. Treatment with platinum-based chemotherapy in the neoadjuvant, adjuvant, or first-line metastatic setting was permitted. Patients who received vinflunine as part of combination therapy or for maintenance were excluded.

The study was conducted according to the Declaration of Helsinki and ethics approval was obtained by the Ethics Committee of Ulm University on 2 July 2020 (reference #216/20).

Patients were divided into two groups according to presence of a prior ICI treatment (post-ICI cohort vs. ICI-naïve cohort). All patients were treated and monitored according to local clinical practice. Outcomes of interest were ORR, clinical benefit rate (CBR), OS, and progression-free survival (PFS). ORR was defined as sum of complete response (CR) and partial response (PR), assessed in accordance with investigator-assessed Response Evaluation Criteria in Solid Tumors (RECIST v1.1). CBR was defined as the sum of CR, PR, and stable disease (SD). Tumor response was assessed by computer tomography or magnetic resonance imaging.

OS was defined as the time period between first vinflunine administration and the date of death from any cause or last follow-up visit. PFS was defined as the time period between first vinflunine administration and the date of radiological disease progression, death, or last follow-up visit. The response was assessed by investigators in each individual institution without a central review process.

Statistical analyses were performed with GraphPad Prism (Version 9.2.0). Summary descriptive statistics were applied to baseline patient characteristics and Mann–Whitney U test, Pearson’s Chi-squared test, and Fisher’s exact test were applied to perform intergroup comparisons, depending on data distribution. OS and PFS were estimated by the Kaplan–Meier method and tested with log-rank tests. Multivariable Cox regression analyses were performed to assess the association of patient characteristics with respect to OS, and univariable binary logistic regression as well as multivariable logistic regression analyses were performed to examine the impact of patient characteristics on response to vinflunine therapy. All tests were two-sided, and *p*-values < 0.05 were considered significant.

## 3. Results

### 3.1. Characteristics of the Study Cohort

A total of 105 patients with mUC of the bladder or upper urinary tract who underwent vinflunine treatment between February 2010 and December 2021 met the study inclusion criteria and were evaluated in this retrospective multicentric cohort study. All included patients were pretreated with gemcitabine/cisplatin or gemcitabine/carboplatin in the neoadjuvant, adjuvant, or first-line metastatic treatment setting. Of them, 61 patients (58.1%) additionally received ICI treatment with atezolizumab, avelumab, nivolumab, nivolumab + ipilimumab, or pembrolizumab for metastatic disease prior to initiation of vinflunine (post-ICI cohort), whereas 44 patients (41.9%) did not (ICI-naïve cohort). Duration of ICI treatment was evaluable in 60 patients of the post-ICI cohort (98.4%), and 25 of 60 patients (41.7%) had short-term ICI treatment of less than 3 months.

The median therapy line vinflunine was applied was a second-line treatment in ICI-naïve (second to fifth-line) and a third-line treatment in post-ICI patients (third to sixth-line). Baseline characteristics were similar in both cohorts, with the exception of a higher rate of bone metastases and pelvic irradiation in the post-ICI cohort. Detailed patient characteristics are depicted in Table 1.

### 3.2. Treatment Exposure and Tolerability

After a median follow-up time of 7.1 months (range: 0.4–69.1), the median number of applied vinflunine cycles (administration every 3 weeks) was 4 (range: 1–48) in the post-ICI cohort und 3 (range: 1–15) within the ICI-naïve cohort. The median vinflunine dose at treatment initiation was 268 vs. 288 mg/m^2^ body surface, respectively. Dose reductions due to adverse events were necessary in 18.0% of the patients pretreated with an ICI and in 11.4% among the ICI-naïve cohort. Grade 3–4 adverse events, according to Common Terminology Criteria for Adverse Events (CTCAE) classification [14], occurred in 30.0% in the post-ICI cohort and in 38.1% in the ICI-naïve cohort. At data cutoff, 44 (100%) of the ICI-naïve patients and 56 (91.8%) of the post-ICI patients had discontinued treatment. Based on investigator assessment, the most common reasons for treatment discontinuation were disease progression (*n* = 82), death (*n* = 6), treatment-related adverse events (*n* = 8), or the patient’s wish (*n* = 4).

### 3.3. Efficacy

#### 3.3.1. Best Overall Response

For response assessment, 49 of 61 patients (80.3%) in the post-ICI cohort and 32 of 44 patients (72.7%) in the ICI-naïve cohort were able to be evaluated for radiographic response assessment. In the overall population, 16 of 81 patients had partial or complete response following vinflunine treatment, corresponding to an ORR of 19.8%. Furthermore, 11 of 49 patients (22.4%) in the post-ICI cohort and 5 of 32 ICI-naïve patients (15.6%) had a radiographic response to vinflunine treatment. The difference in ORR between post-ICI and ICI-naïve was statistically not significant (*p* = 0.451).

A total of 33 out of 81 patients had at least stable disease as the best overall response, corresponding to a CBR of 40.7%. Stable disease or radiographic response were observed in 25 out of 49 patients (51.0%) in the post-ICI cohort and 8 out of 32 (25.0%) in the ICI-naïve cohort. The difference in CBR between post-ICI and ICI-naïve was statistically significant (*p* = 0.020; Table 2).

#### 3.3.2. Survival

Median PFS was 2.57 months (95% CI 2.07–3.68 months) in the total cohort. In the post-ICI cohort, PFS was slightly longer with 3.09 months (95% CI 2.17–4.70) than in the ICI-naïve cohort with 2.14 months (95% CI 1.71–2.76), corresponding to a hazard ratio of 0.726 (95% CI 0.484–1.090). The difference was not statistically significant (*p* = 0.105). At 3 months, the PFS rate was 52.5% among post-ICI patients and 34.1% among ICI-naïve patients. During follow-up, 50 out of 61 patients in the post-ICI-cohort and 43 out of 44 patients in the ICI-naïve cohort had died.

Median OS was 8.78 months in the post-ICI cohort and 5.72 months in the ICI-naïve cohort, corresponding to a hazard ratio of 0.861 (95% CI 0.571–1.299; *p* = 0.467). The 6-month OS rate was 58.6% within the post-ICI cohort and 50.0% within the ICI-naïve cohort. The 9-month OS rate was 47.4% and 34.1%, respectively.

**Figure 1:**
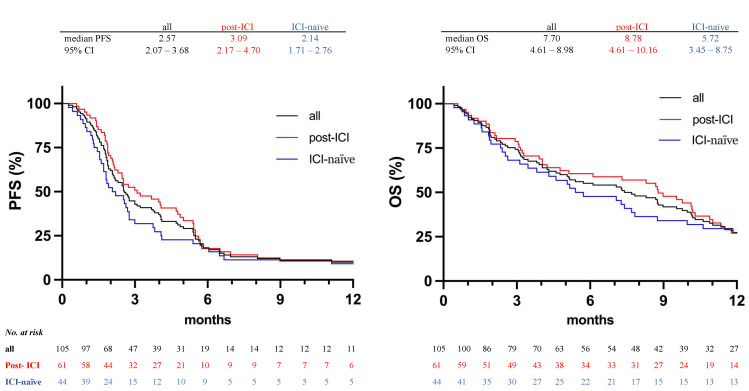
Kaplan–Meier curves for PFS and OS in both cohorts.

Kaplan-Meier curves for PFS and OS in both cohorts are shown in Figure 1, and further details on survival rates under vinflunine treatment are given in Table 3.

#### 3.3.3. Clinical Predictors of Treatment Efficacy of Vinflunine

Univariable binary logistic regression analyses in all patients showed a significantly higher probability of considerable treatment effects (SD, PR, or CR) with an odds ratio of 3.900 (95% CI 1.494–10.99; *p* = 0.007) when prior ICI therapy was performed. Previously evaluated risk factors for second-line chemotherapy by Bellmunt et al. were not associated with the treatment effect (see Table 4).

Since ICI pretreatment was significantly associated with bone metastases and prior pelvic irradiation (see Table 1), we performed multivariable logistic regression after adjustment for these two variables. At this ICI pretreatment was also associated with a considerable treatment effect (OR 6.895; 95% CI 2.139–25.66; *p* = 0.002; see Table 5).

When univariable binary logistic regression analysis of considerable treatment effect was performed only within the post-ICI cohort, no statistically significant association was revealed. Of note, patients who received immunotherapy for less than 3 months showed a 3.1-fold higher probability of considerable treatment effect compared to those with ICI therapy for at least 3 months (OR 3.143; 95% CI 0.8590–13.45; *p* = 0.097).

#### 3.3.4. Clinical Predictors for the Risk of Death

The risk of death was reduced by 35.5% when ICI pretreatment was performed, though statistical significance was not reached (HR 0.6451; 95% CI 0.3983–1.045; *p* = 0.074).

A poor performance status of ECOG 1 or higher and a low hemoglobin level of less than 10 g/dL were significantly associated with a higher risk of death (HR 2.248 and 1.737, respectively; see Table 6).

## 4. Discussion

To this day, there is still significant uncertainty regarding the best treatment course in platinum-refractory mUC patients pretreated with ICI. While the most reliable data are available for the antibody–drug conjugate enfortumab vedotin [15], there is a lack of knowledge in regard to other treatment options, highlighted in a recently published systemic review by Deiniger et al. [12]. In Europe, also after the recent EMA-approval for enfortumab vedotin, vinflunine is still a standard of care after failure of sequential platinum-based chemotherapy and ICI treatment [2,16].

This study represents, to the best of our knowledge, the first comprehensive analysis of the efficacy of single-agent chemotherapy with vinflunine in patients with mUC with or without ICI pretreatment. Our data show that vinflunine has a measurable clinical benefit in a third-line setting in patients with prior platinum-based chemotherapy and ICI pretreatment. The ORR of 22.4% is more than twofold higher, and the OS of 8.78 months is more than 20% higher than in the historical cohort of second-line patients in the pivotal trial [9]. The absence of statistical significance is most likely due to relatively small patient cohort and few survival outliers within the ICI-naïve cohort. Our findings speak for a sustained clinical activity of vinflunine in a real-world setting, even in later treatment stages. Corresponding data were recently published by Bersanelli et al., who could show an ORR of 18.5% for salvage chemotherapy after progression to previous platinum-based chemotherapy and ICI [17]. Median PFS and OS in the Italian multicenter study were 3 and 9 months, respectively, although of note, in contrast to our analysis, salvage chemotherapy included monotherapy with vinflunine, gemcitabine, or taxanes as well as cisplatin-based combinations. On the basis of these promising results, it was postulated by the authors that ICI pretreatment would have provided a “time out” to the disease.

This finding is supported by our comparisons of post-ICI with ICI-naïve patients. The baseline characteristics did not differ between the two cohorts, with the exception of worse prognostic parameters in the post-ICI cohort (higher incidence of bone metastases and prior pelvic irradiation). The data indicate maintained efficacy of third-line vinflunine in the era of immunotherapy, which appears to be comparable to that of reported clinical trials, if not slightly superior [9,10]. This is supported by the finding, that the remarkable CBR of 51.0% within the post-ICI cohort was around twofold higher compared with ICI-naïve-patients (25.0%).

It is noteworthy that the univariable binary logistic regression analyses revealed that the presence of previous ICI treatment was the only significant predictor of considerable therapy response with vinflunine, while previously evaluated negative prognostic factors by Bellmunt et al. [13] (poor performance status, presence of liver metastases, and low hemoglobin level) were not statistically significant. We did, however, see that a poor performance status of ECOG 1 or higher as well as a low hemoglobin level of less than 10 g/dl were significantly associated with a higher risk of death. Consequently, ICI pretreatment should be considered as a key prognostic factor in patients receiving third-line vinflunine.

A higher tumor sensitivity to chemotherapy after exposure to ICI has also been described for patients with non-small cell lung cancer, squamous cell carcinoma of the head, neck cancer, and malignant melanoma [18,19,20,21]. It is hypothesized that ICI treatment leads to a change in the tumor microenvironment with accumulation of activated CD8^+^ T cells [19]. These T cells might be further activated by chemotherapy, which has been found to have not only immunosuppressive effects, but also immune-activating properties [22]. Taken together, the sequence of ICI-therapy followed by chemotherapy could have the potential to act antineoplastic by both, the direct cytotoxity of chemotherapy along with immune-dependent anti-neoplastic effects, which derive from the interplay of the sequential administration of both therapy regimens [19]. This hypothesis should be investigated by more detailed longitudinal studies of the immune status in mUC patients sequentially treated with ICI and chemotherapy.

Besides clinical parameters, there is still an unmet medical need of valuable biomarkers that could optimize the individual therapy algorithm of each mUC patient in a rapidly changing treatment landscape [23,24]. In a recently published study by Bernardini et al., the genomic landscape of vinflunine response was investigated [25]. It was demonstrated that tumors refractory to vinflunine showed immune signatures potentially associated with response to ICI. That leads to the hypothesis that in patients rapidly progressing during ICI therapy biological tumor characteristics might be prevalent that are associated with the tumor response to vinflunine. This matches with our analysis where patients with short-term ICI pretreatment for less than 3 months showed a higher probability of response to subsequent vinflunine therapy compared to those with long-lasting ICI pretreatment, indicating a major clinical benefit in these patients. In this context, the rare phenomenon of pseudo-progression during ICI treatment must also be considered [5]. Hopefully, future studies in this highly relevant field can improve oncologic decision making with regard to personalized selection of the most suitable tumor therapy. Furthermore, the sequential therapy of vinflunine and the upcoming antibody–drug conjugate enfortumab vedotin must be evaluated in further clinical trials, since their mechanism of action as a microtubule inhibitor is quite similar.

The limitations of our study are the moderate sample size of our study, biases that may derive from the retrospective study design, and the different timespans in which patients were exposed to vinflunine in the two cohorts. Furthermore, the patients’ sex was not reported, which could have influenced the outcome.

## 5. Conclusions

Our findings confirm the efficacy of vinflunine in unselected platinum- and ICI-refractory patients and support the use of vinflunine in the changing treatment paradigm of relapsed mUC. Patients who received treatment with both platinum-based chemotherapy and ICI have improved outcomes when receiving vinflunine compared to patients who have not received ICI. Especially patients who are rapidly progressing during ICI therapy may experience a major clinical benefit. Further study is needed to elucidate the mechanism surrounding our intriguing hypothesis.

## Figures and Tables

**Table 1:** Patient characteristics at baseline.

Patient Characteristics at Baseline	Post-ICI *n =* 61 (58.1%) April 2016–December 2021	ICI-Naïve *n* = 44 (41.9%) February 2010–May 2019	*p*
Age (years)			
median	66.5	66.3	0.9549
IQR range	14.65 49–87	11.97 51–84	
Site of primary tumor			
lower tract	82.0% (50)	72.7% (32)	0.2587
upper tract	18.0% (11)	27.3% (12)	
Therapy line			
median mean SD	3 3.2 0.68	2 2.36 0.61	<0.0001
range	3–6	2–5	
Period from initial diagnosis of MIBC ^1^ or primary mUC to first Vinflunine administration (months)			
median IQR	24.4 33.0	15.5 30.4	0.0333
range	6.6–156.6	3.3–128.7	
Metastatic site			
lymph node	86.9% (53)	84.1% (37)	0.6864
bone	57.4% (35)	22.7% (10)	0.0341
pulmonary	44.3% (27)	36.4% (16)	0.4167
visceral liver	63.9% (39) 34.4% (21)	63.6% (28) 40.9% (18)	0.9750 0.4976
Negative prognostic factors by Bellmunt et al. [13]			
median	1	1	0.9855
mean SD	1.28 0.90	1.27 0.95	
Hb < 10 g/dL ^2^	24.6% (15)	22.7% (10)	0.8250
ECOG ^3^			
0 1 2 3 median mean SD	31.1% (19) 49.2% (30) 16.4% (10) 3.3% (2) 1 0.92 0.78	36.4% (16) 52.3% (23) 9.1% (4) 2.3% (1) 1 0.77 0.71	0.5759 0.7545 0.3859 >0.9999 0.3638
Renal function (GFR; ml/min) ^4^			
median IQR range	53 28.0 22–99	58 25.5 27–133	0.3551
Prior cystectomy or nephroureterectomy	63.3% (38)	56.8% (25)	0.5018
Neoadjuvant therapy	11.7% (7)	2.3% (1)	0.1340
Prior irradiation of the pelvis	23.3% (14)	6.8% (3)	0.0314

^1^ MIBC = muscle invasive bladder cancer. ^2^ Hb = hemoglobin level. ^3^ ECOG = Eastern Cooperative Oncology Group. ^4^ GFR = glomerular filtration rate.

**Table 2:** Treatment response.

End Point	All	Post-ICI	ICI-Naïve	*p*
No. of included patients	105	61	44	
Overall response in evaluable patients				
No. of patients	81	49	32	
Complete response				
No. of patients	1	1	0	
%	1.2	2.0	0	
Partial response				
No. of patients	15	10	5	
%	18.5	20.4	15.6	
Stable disease				
No. of patients	17	14	3	
%	21.0	28.6	9.4	
Overall response rate				0.4508
No. of patients	16	11	5	
%	19.8	22.4	15.6	
95% CI,%	12.5–29.7	13.0–35.9	6.9–31.8	
Clinical benefit rate				0.0198
No. of patients	33 40.7 30.1–51.6	25 51.0 37.5–64.4	8 25.0 13.3–42.1	
% 95% CI,%				

**Table 3:** PFS and OS rates after 3, 6, 9, and 12 months.

Survival	All	Post-ICI (*n* = 61)	ICI-Naïve (*n* = 44)
PFS after 3 months (%)	44.8 (47/105)	52.5 (32/61)	34.1 (15/44)
PFS after 6 months (%)	18.4 (19/103)	16.9 (10/59)	20.4 (9/44)
PFS after 9 months (%)	11.7 (12/103)	11.9 (7/59)	11.4 (5/44)
PFS after 12 months (%)	10.7 (11/103)	10.2 (6/59)	11.4 (5/44)
OS after 3 months (%)	75.2 (79/105)	80.3 (49/61)	68.2 (30/44)
OS after 6 months (%)	54.9 (56/102)	58.6 (34/58)	50.0 (22/44)
OS after 9 months (%)	41.6 (42/101)	47.4 (27/57)	34.1 (15/44)
OS after 12 months (%)	27.0 (27/100)	25.0 (14/56)	29.5 (13/44)

**Table 4:** Univariable binary logistic regression analysis of considerable treatment effect (SD, PR, or CR) in all patients.

Variable	OR	95% CI	*p*
ICI pretreatment	3.900	1.494–10.99	0.0071
age > 70 years	0.6293	0.2423–1.586	0.3310
GFR ^1^ < 60 mL/min	0.6923	0.2785–1.704	0.4241
lymph node disease only	0.6705	0.1882–2.166	0.5134
liver metastases	0.7292	0.2730–1.888	0.5190
prior pelvic irradiation	0.6944	0.1946–2.249	0.5517
pulmonary metastases	1.254	0.5035–3.130	0.6257
Hb ^2^ < 10 g/dL	1.224	0.4272–3.479	0.7027
initial dose of 320 mg/m^2^ body surface	1.588	0.8738–2.969	0.7621
bone metastases	0.8942	0.3538–2.232	0.8109
Upper tract urothelial carcinoma	0.8815	0.2861–2.603	0.8206
Bellmunt risk factors ≥ 1	0.9832	0.3880–2.468	0.9712
ECOG ^3^ ≥ 1	0.9890	0.3976–2.475	0.9810

^1^ GFR = glomerular filtration rate. ^2^ Hb = hemoglobin level. ^3^ ECOG = Eastern Cooperative Oncology Group.

**Table 5:** Multivariable logistic regression analysis of considerable treatment effect (SD, PR, or CR) in all patients.

Variable	OR	95% CI	*p*
ICI pretreatment	6.895	2.139–25.66	0.0021
bone metastases	0.4234	0.1076–1.276	0.1557
prior pelvic irradiation	0.4824	0.1213–1.780	0.2820

**Table 6:** Multivariable Cox regression analyses of OS in all patients.

Variable	HR	95% CI	*p*
ECOG ^1^ ≥ 1	2.248	1.365–3.825	0.0020
Hb ^2^ < 10 g/dL	1.737	1.013–2.897	0.0386
liver metastases	1.565	0.9802–2.483	0.0581
ICI pretreatment	0.6451	0.3983–1.045	0.0742
bone metastases	1.255	0.7529–2.103	0.3857
initial dose of 320 mg/m^2^ body surface	1.129	0.7103–1.777	0.6021
GFR ^3^ < 60 mL/min	0.9126	0.5737–1.454	0.6987
age > 70 years	0.9323	0.5784–1.483	0.7700
prior pelvic irradiation	1.009	0.5512–1.764	0.9746

^1^ ECOG = Eastern Cooperative Oncology Group. ^2^ Hb = hemoglobin level. ^3^ GFR = glomerular filtration rate.

## Data Availability

The data that support the findings of this study are available from the corresponding author upon reasonable request.
